# A systematic review on how primary care electronic medical record data have been used for antimicrobial stewardship

**DOI:** 10.1017/ash.2024.499

**Published:** 2025-01-24

**Authors:** Ron Cheah, Caroline Chen, Daniel Capurro, Jo-Anne Manski-Nankervis, Vlada Rozova, Karin Thursky

**Affiliations:** 1 The National Centre for Antimicrobial Stewardship, Department of Infectious Diseases, Melbourne Medical School, The University of Melbourne, Melbourne, Victoria, Australia; 2 RMH Guidance Group, The Royal Melbourne Hospital, Melbourne, Victoria, Australia; 3 The Peter Doherty Institute for Infection and Immunity, Melbourne, Victoria, Australia; 4 School of Computing and Information Systems, Faculty of Engineering and Information Technology, The University of Melbourne, Melbourne, Victoria, Australia; 5 Centre for the Digital Transformation of Health, Faculty of Medicine, Dentistry, and Health Sciences, The University of Melbourne, Melbourne, Victoria, Australia; 6 Primary Care and Family Medicine, Lee Kong Chian School of Medicine, Nanyang Technological University of Singapore, Singapore, Singapore; 7 Department of General Practice and Primary Care, The University of Melbourne, Parkville, Victoria, Australia; 8 National Health and Medical Research Council National Centre for Infections in Cancer, Sir Peter MacCallum Department of Oncology, University of Melbourne, Parkville, Victoria, Australia

## Abstract

**Objective:**

This systematic review aims to synthesize evidence from current literature to describe how Electronic Medical Record (EMR) primary care data have been used for antimicrobial stewardship activities internationally.

**Design:**

Systematic literature review.

**Methods:**

We searched Cumulative Index to Nursing and Allied Health Literature (CINAHL), PubMed, Embase, Scopus, and Web of Science from January 1, 2013 to September 23, 2023 to retrieve studies that included concepts of “antimicrobial stewardship,” “primary care,” and “electronic medical records.” We used narrative synthesis to classify and interpret results. Data were grouped and tabulated by similar themes and concepts, including strengths, facilitators, barriers, and limitations for antimicrobial stewardship.

**Results:**

A total of 265 articles were identified from the initial search, of which 34 full-text articles from 10 countries met all criteria and were included in the review. Six categories of EMR data use were identified from the studies, these were for: assessing antimicrobial prescribing quality, measuring the effectiveness of an intervention, analyzing antimicrobial prescribing trends, assessing patient and provider characteristics in prescribing, evaluating novel tools or measures, and measuring specific conditions and outcomes. Facilitators of use of EMR data were generally well-described across the studies reviewed; however, barriers were not. Barriers described were centered around EMR system design and technical challenges in data extraction. Completeness of EMR data was the most consistently described limitation.

**Conclusions:**

Our study highlights the range of uses of EMR data in supporting AMS in primary care internationally, and its strengths, facilitators, and barriers to use.

## Introduction

Antimicrobials are essential medicines used to treat and prevent infectious diseases. These medicines enable many life-saving medical interventions such as surgical procedures, cytotoxic chemotherapy, and safe administration of immunosuppressants. Unfortunately, they are overused on a global scale, driving one of the greatest threats to humanity, known as antimicrobial resistance (AMR).^1^ The impact and implications of AMR are far-reaching; in addition to mortality, AMR contributes to prolonged treatment times, increased healthcare costs, unnecessary hospitalizations for conditions generally managed in the community,^2^ and has hindered several countries in reaching their sustainable development goals.^3^

Addressing the issue of AMR necessitates immediate and coordinated actions. National action plans have been developed that incorporate antimicrobial stewardship (AMS) strategies in response to the rising threats of AMR. AMS is a coordinated set of strategies aimed at understanding antimicrobial use through quality and quantity of use surveillance, optimizing use by enhancing prescription appropriateness through interventions such as audit and feedback, and minimizing adverse effects associated with use. This is particularly critical in the primary care setting, where most antimicrobial prescribing occurs.^4–6^ Primary care is defined as the “health care people seek first in their community,” this typically includes general practitioners, pharmacists, and other health professionals.^7,8^ Despite its importance, AMS in primary care is often under-resourced and insufficiently implemented in many countries.

The advent of electronic medical records (EMRs) has signaled a positive change in improving health care. EMRs provide clinicians with well-organized, linked information in a format that is easy to search - functionality not previously possible with paper records. This has led to an improvement in chronic disease management and prevention, and attainment of screening targets.^9,10^ Large, population-wide databases have become important resources for public health research,^11^ with major projects throughout the world. Therefore, similarly, these data could be valuable for supporting AMS efforts at scale.

This systematic review aims to explore the use of primary care EMR data for supporting AMS internationally, an activity not previously undertaken. The objectives are to identify the types of studies and interventions performed with these data and their findings, investigate reported data quality issues, and identify facilitators and barriers to its use. Learnings can be applied to improve existing systems and to inform the design of future EMR systems and processes across various settings to better facilitate AMS in primary care.

## Methods

This review was registered on The International Prospective Register of Systematic Reviews (PROSPERO) on the 14^th^ of September 2023 (CRD42023460384) and followed the Preferred Reporting Items for Systematic Reviews and Meta-Analyses (PRISMA) guidelines.^12^

### Eligibility criteria

All studies conducted in the primary care setting relating to primary care EMR data use for AMS interventions were included. In this study, we use the terms EMR and electronic health record (EHR) interchangeably as it is applicable in this context, although not synonymous. AMS interventions includes any or all of the following activities^13^: (i) acting on antimicrobial use and appropriateness audit results for continuous quality improvement, (ii) reviewing antimicrobial prescribing use, and ensuring appropriate documentation of indication, active ingredient, dose, frequency, route of administration, intended duration or review plan, and adverse reactions in a patient’s healthcare record,^1^ (iii) using surveillance data on antimicrobial consumption, use, and resistance to support appropriate prescribing, (iv) evaluating AMS program performance, identifying areas for improvement, and act to improve appropriateness of antimicrobial prescribing and use, (v) reporting to clinicians and governing bodies on compliance with the AMS policy and guidance, areas of action for AMR, areas of action to improve appropriateness of prescribing and compliance with current evidence-based guidelines or resources on antimicrobial prescribing, and the health service organization’s performance over time.

### Exclusion criteria

Studies were excluded if they (i) contained incomplete or unclear data, (ii) were review articles, meta-analyses, gray literature, editorials, opinion pieces, commentaries, conference proceedings, or posters, or (iii) were published in any language other than English.

### Search strategy

Relevant articles were identified by a broad search of the following electronic databases: Cumulative Index to Nursing and Allied Health Literature (CINAHL), PubMed, Embase, Scopus, and Web of Science for articles between January 1, 2013 and September 23, 2023. The strategy included search terms to retrieve concepts of AMS, primary care, and EMR.

“Antimicrobial stewardship,” “primary care,” and “electronic medical records” are referred to by different terms depending on country and context. Common aliases for antimicrobial stewardship include “antibiotic stewardship”^14^ and the acronyms “AMS”^15^ or “ASP”;^16^ other terms referring to primary care include “general practice” or the acronym “GP,”^17^ “family practice” or “family medicine”;^18^ and “electronic medical records” are often referred to as its acronym “EMR.” Medical subject headings (MeSH) terms were used in addition to text words to increase search sensitivity. The final search strategy is available in supplementary Table S1.

#### Screening

Two independent reviewers (RC and CC) used Covidence® systematic review software to screen titles and abstracts for eligibility following deduplication. RC, DC, KT, and JMN collated a list of specific terms to be highlighted for reviewers to consider for potential inclusion or exclusion (supplementary Table S2). Reviewers manually screened articles based on these and sorted each into the following categories: (a) meets eligibility criteria (b) does not meet eligibility criteria, and (c) unclear if it meets eligibility criteria. Full-text screening was performed on the articles in categories (a) and (c) by the same two reviewers (RC and CC). Any disagreements between the screening authors were resolved by discussion with a third review author (KT, DC, or JMN). Only publications passing both abstract and full-text screens were included.

### Study quality assessment

The final full-text studies deemed suitable for inclusion were further appraised for quality and risk of bias independently by two reviewers (RC and CC), with any disagreements resolved by discussion. A third reviewer was nominated to adjudicate any disputes (KT, DC, or JMN). The Joanna Briggs Institute suite of critical appraisal tools was used to perform these assessments, where the tool most relevant to the type of study being appraised was utilized. The overall risk of bias was assessed as low for all the included studies, with no significant concerns identified.

### Data extraction and synthesis

A narrative synthesis was employed to classify and interpret results. Reviewers RC and CC independently grouped and tabulated data based on relevant similarities in themes and concepts, including strengths and facilitators, as well as barriers and limitations to the use of EMR for antimicrobial stewardship activities in primary care. “Strengths” in this context referred to characteristics inherent to the data sources, while “facilitators” were defined as external factors that facilitated the effective use of these data to support AMS. Similarly, “barriers” were any external factors that were perceived to hinder effective data use in this context, and “limitations” were framed as issues that could potentially impact data quality. Results were reported following PRISMA guidelines.^12^

#### Ethics

Ethics approval was not required.

## Results

### Characteristics of included studies

**Figure 1.**
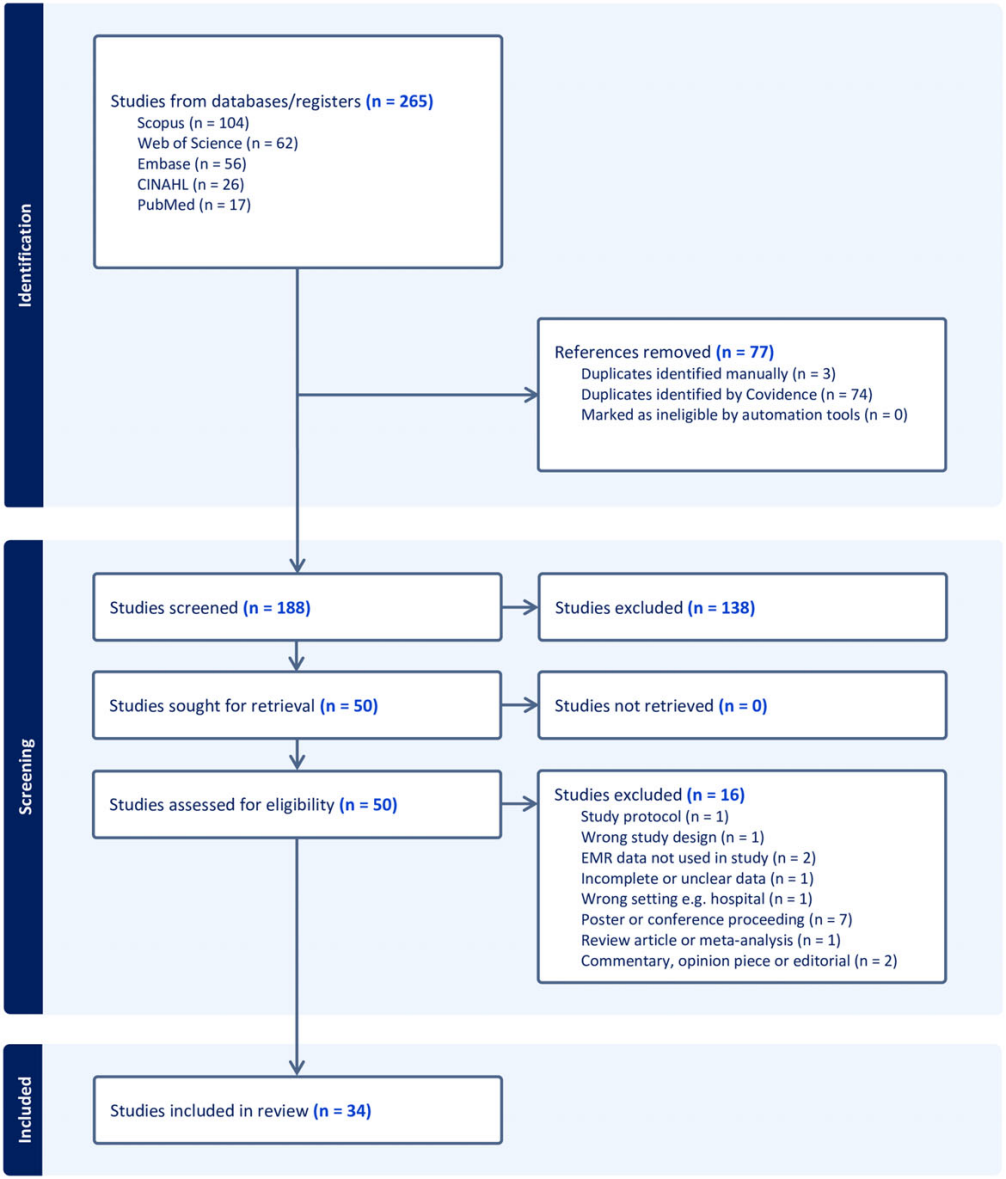
PRISMA 2020 flow diagram.

The literature search resulted in a total of 265 articles. After deduplication, a set of 188 articles underwent title and abstract screening where 138 articles were excluded as deemed irrelevant. The remaining 50 articles were included in a full-text review, where a further 16 articles were excluded. A final total of 34 full-text articles that met all criteria were included for review (Figure 1).

Most studies were from North America (USA, n = 15; Canada, n = 4), followed by Europe (The United Kingdom, n = 7; The Netherlands, n = 2; France, n = 1; Spain, n = 1; Switzerland, n = 1) and the remaining from Africa (Ghana, n = 1), Asia (China, n = 1), and Oceania (Australia, n = 1). Among these, there were 19 cohort studies, 5 cross-sectional studies, 5 quasi-experimental studies, 2 randomized controlled trials, 1 quality improvement study, 1 descriptive observational study, and 1 mixed-methods randomized controlled trial and cohort study. Twenty-nine unique data sources were identified in these studies; five studies were conducted with data from the Clinical Practice Research Datalink (CPRD)^19–23^ and two obtained data from the same two private family medicine clinics^24,25^

### EMR data for supporting AMS

Six categories of EMR data used for supporting AMS were identified from the studies included in the review. These were, (i) assessing antimicrobial prescribing quality,^23,25–40^ (ii) measuring the effectiveness of an intervention,^25,28,29,34,37,38,40–44^ (iii) analyzing antimicrobial prescribing trends,^22–24,26,27,31,36,37,39,45–52^, (iv) assessing patient and provider characteristics in prescribing^21–24,26,27,31,32,36,38,39,47,49,51,52^ (v) evaluating novel tools or measures^33,53^, and (vi) measuring specific conditions and outcomes.^19,23,35,48,50,52^ The specific conditions and outcomes measured were: serious infection rates due to lower antibiotic prescribing, impetigo incidence, treatment and recurrence, prevalence and documentation quality of beta-lactam allergies, changes in antibiotic prescribing for different patient demographics and indications over time, male urinary tract infection prevalence, and pre- and post-pandemic respiratory tract infection (RTI) presentations. These are described and summarized in Table 1.

**Table 1.** Use categories matched to study outcomes of interest and key findings

Use category	Reference	Country	Outcome(s) of interest	Key findings
Analyzing antimicrobial prescribing trends	Adekanmbi 2020^45^	UK	Association between socioeconomic status (SES) and antibiotic prescribing.	Higher rates of antibiotic prescribing in individuals in the most deprived SES quintile compared to those in the least deprived quintile. Disparities persist even after accounting for potential confounding factors.
	Brown 2019^26^	USA	Fluoroquinolone (FQ) prescribing rates between different primary care clinic types.	FQ were prescribed more frequently at nonacademic clinics compared to academic clinics.
	Chandra Deb 2022^27^	USA	Antibiotic usage rates in adults for pharyngitis without a positive test for group A Streptococcus, uncomplicated acute bronchitis, and nonspecific acute upper respiratory tract infection.	Acute bronchitis had the highest unnecessary prescribing rate, followed by acute respiratory infections, acute upper respiratory infections, and pharyngitis.
	Grigoryan 2017^24^	USA	Antibiotic prescribing patterns for uncomplicated acute bronchitis.	Antibiotics were prescribed in approximately a third of visits for uncomplicated acute bronchitis. Broad-spectrum antibiotics constituted the majority of all prescriptions, with macrolides (mostly azithromycin) being the most common. Other antibiotics prescribed included amoxicillin-clavulanate, fluoroquinolones, and amoxicillin. Azithromycin was prescribed for an average of 5.1 days, while amoxicillin-clavulanate was prescribed for 9.8 days.
	Gulliford 2020^46^	UK	Frequency of serious bacterial infections at family practices with lower antibiotic prescribing rates.	No evidence indicating that reduced antibiotic prescribing in family practices leads to higher rates of serious bacterial infections.
	Hawes 2018^31^	Australia	Australian General Practitioner antibiotic prescribing patterns.	Cefalexin was consistently the most frequently prescribed antibiotic throughout the 5-year study period. Seasonal fluctuations were observed in the prescribing patterns of amoxicillin-clavulanate, roxithromycin, doxycycline, clarithromycin, cefaclor, erythromycin, and phenoxymethylpenicillin.
	Kitano 2021^47^	Canada	Association between total and unnecessary antibiotic use.	Strong correlation between the total antibiotic prescribing volume and unnecessary prescribing rate, suggesting that total antibiotic volume could be used as a surrogate of unnecessary prescribing rate. Significant variability in unnecessary antibiotic prescribing rates were observed and this was largely unexplained by practice- or physician-level factors.
	Loadsman 2019^48^	The Netherlands	Impetigo incidence, treatments, and recurrence.	Topical fusidic acid was the most commonly prescribed initial treatment followed by oral flucloxacillin. Among 1761 impetigo episodes, 87.6% received GP-prescribed treatment. Most treated episodes received treatment on the day of diagnosis, with a small proportion (less than 2%) receiving treatment within 7 days.
	Martínez-González 2020^49^	Switzerland	Patterns of antibiotic prescribing and associated factors.	Broad-spectrum penicillins saw a consistent increase in prescriptions, while quinolones declined steadily over the study period (2008 to 2020). Antibiotic prescriptions varied significantly by month, peaking during cold seasons and dropping in summer. Overall mean number of antibiotic prescriptions per patient was 1.65. Most common indications for antibiotics: respiratory tract infections, urinary tract infections, and skin infections. Top prescribed antibiotics: amoxicillin plus beta-lactamase inhibitors, ciprofloxacin, and clarithromycin.
	Owusu 2022^36^	Ghana	Rates of compliance with standard treatment guidelines among adults with uncomplicated urinary tract infections (UTI).	Six out of ten patients received antibiotics as specified in guidelines for dose, frequency, and duration.
	Ray 2021^37^	USA	Guideline-concordant antibiotic management for acute respiratory tract infection in children via telemedicine integrated within a pediatric primary care service.	Guideline-concordant antibiotic management for sinusitis and viral acute respiratory tract infections during telemedicine visits increased over the study period.
	Rockenschaub 2020b^22^	UK	Association between the onset of comorbidity and antibiotic prescribing.	Antibiotic prescribing rates rise significantly four to nine months before COPD, heart failure, and asthma diagnoses, however this declines to baseline levels within 2 months post-diagnosis. This suggests that respiratory symptoms may sometimes be misdiagnosed as respiratory tract infections which can lead to increased antibiotic prescribing. A similar but less marked trend of increased prescribing rate was observed for diabetes. Rates of prescribing for patients with vascular conditions however, increased immediately before diagnosis but remained higher than baseline afterwards.
	Singer 2018^39^	Canada	Frequency of potentially inappropriate antimicrobial prescribing.	Approximately a fifth of primary care visits for common infections resulted in potentially inappropriate antimicrobial prescriptions. Among visits where bacterial infections were diagnosed, 37.8% received potentially inappropriate antimicrobials, and 19.6% received antimicrobials for durations outside guideline-based ranges. 15.9% of visits for viral infections included antimicrobial prescriptions.
	Soudais 2021^50^	France	Trends of prescribed treatments of male UTIs	FQs were the most prescribed antibiotic for this condition and this was in alignment with national guidelines.
	Sun 2019^23^	UK	Antibiotic prescribing patterns and trends for different age groups, genders, and indications over time.	The rate of antibiotic prescriptions and proportion of patients receiving antibiotics declined consistently over the study period. Antibiotic prescriptions that were not associated with medical codes showed the slowest rate of decline, potentially further identifying this category of prescriptions representing a suboptimal standard of clinical practice. Respiratory conditions were the most common indication for antibiotics and showed the greatest rate of decline.
	Wang 2023^51^	China	Antibiotic prescribing patterns among patients with acute respiratory infections in rural primary healthcare facilities.	The majority of patients diagnosed with acute respiratory infection were prescribed at least one antibiotic. 37.82% of these prescriptions included multiple antibiotics, with 55.29% being parenteral. Watch group antibiotics were prescribed more frequently than Access^70^ group antibiotics.
	Wong 2023^52^	Canada	Pre- and post-pandemic respiratory tract infection (RTI) antibiotic prescribing rates	RTI consultation rates decreased from pre- to post-pandemic with a corresponding decrease in antibiotic prescribing rates. This represents 750,000 fewer patients prescribed potentially avoidable antibiotics for RTI in primary care. Significant drop in antibiotic prescriptions for RTIs across all deprivation levels. Baseline trend showed patients with less deprivation had a higher reduction rate in antibiotic prescriptions over time.
Measuring the effectiveness of an intervention or service	Blair 2023^41^	UK	Effectiveness of a user-friendly, multifaceted intervention for children with respiratory tract infections.	Implementation of a multifaceted antibiotic stewardship intervention for children with respiratory tract infections did not reduce overall antibiotic dispensing or increase hospital admissions for these infections. Subgroup analyses suggested minor, non-clinically significant reductions in antibiotic prescribing rates in certain contexts.
	Foreman 2022^28^	USA	Impact of stewardship-driven order sentences within the outpatient EHR and education on antibiotic prescribing for urinary tract infections and skin and soft tissue infections.	Implementation of antibiotic order sentences significantly improved total guideline-concordant antibiotic prescribing with significant enhancements in appropriate drug selection and duration.
	Frost 2022^29^	USA	Effectiveness of a bundled antibiotic stewardship intervention compared with an EHR-only intervention in reducing prescribing of antibiotics for longer than institution-recommended durations for Acute Otitis Media in children aged 2 to 18 years	Bundled and EHR-only antimicrobial stewardship interventions effectively increased guideline-concordant antibiotic durations in children over the age of 2 with uncomplicated acute otitis media. Uncertain whether variations in implementation sites or specific intervention components contributed to this difference. Neither intervention led to increased rates of treatment failure or recurrence in this patient group.
	Gerber 2013^42^	USA	Effect of an antimicrobial stewardship intervention on antibiotic prescribing for pediatric outpatients with acute respiratory tract infections.	Broad-spectrum antibiotic prescribing decreased significantly in both intervention and control groups over 12 months. The difference of differences between groups was 6.7%. Reduction in broad-spectrum antibiotics also observed for specific acute respiratory tract infections. No significant change in antibiotic prescribing for viral infections. Intervention led to nearly halved broad-spectrum antibiotic prescribing in children during acute primary care visits.
	Giancola 2020^30^	USA	Effect of a system-based intervention on adherence to guideline-recommended durations of therapy for uncomplicated cystitis in the outpatient setting.	Immediate and sustained improvements in adherence were observed following implementation of intervention. Adherence rates increased notably for nitrofurantoin and trimethoprim-sulfamethoxazole, but not for ciprofloxacin.
	Grigoryan 2021^25^	USA	Impact of a multifaceted stewardship intervention on adherence to evidence-based practice guidelines for the treatment of uncomplicated cystitis.	Difference-in-differences analysis showed a significantly greater increase in adherence at the intervention site compared to the control site.
	Gulliford 2019^20^	UK	Impact of a complex multicomponent intervention to improve antimicrobial stewardship for respiratory tract infection (RTI) management.	Antibiotic prescribing for RTIs was reduced by electronically delivered interventions. Prescribing was reduced for adults aged 15–84 years, but not for children or people over the age of 84 years. Findings suggest that future strategies for antimicrobial stewardship should employ stratified interventions that are tailored to specific age groups.
	Llor 2022^43^	Spain	Effect of delayed antibiotic prescriptions on antibiotic consumption of antibiotics.	The strategy of delayed antibiotic prescribing resulted in a reduction in antibiotic use. Prescriptions were never obtained in approximately 40% of cases. Out of 76 patients who obtained the delayed prescription, 36 declared to have filled the medication the same day of the visit. Only 12 patients of this cohort obtained the medication based on the instructions given by the doctors.
	May 2022^44^	USA	Impact of switching from rapid antigen detection tests (RADTs) to point-of-care polymerase chain reaction testing on antibiotic use in patients with pharyngitis symptoms.	Intervention did not affect overall antibiotic prescription rates. However, in patients testing negative at point of care, there was a significant reduction in antibiotic prescriptions compared to standard of care RADTs. During the control period, fewer cultures were performed with negative RADTs, sometimes resulting in no culture sample submission. Most clinics showed a decrease in antibiotic prescriptions, while a minority saw increases instead.
	McCormick 2020^34^	USA	Impact of a pharmacist-led Ambulatory AMS team on prescribing patterns focused on uncomplicated cystitis and pyelonephritis.	Pharmacist-led intervention improved clinician antibiotic selection, dose, and duration for uncomplicated cystitis and pyelonephritis Post-intervention, no increase in re-treatment rates despite shorter therapy durations was observed
	Ray 2021^37^	USA	Acute respiratory tract infection antibiotic management for children via telemedicine integrated within a pediatric primary care service.	Telemedicine integrated into pediatric primary care practices improved guideline-concordant antibiotic management for acute respiratory tract infections.
	Vanstone 2022^40^	Canada	Effect of audit and feedback intervention on antimicrobial prescriptions indication documentation.	The audit and feedback intervention proved to be a useful strategy for improving antimicrobial indication documentation.
Assessing Antimicrobial Prescribing Quality	Brown 2019^26^	USA	Appropriateness of fluoroquinolone prescribing in different types of primary care clinics.	The average duration of FQ therapy was 7.1 days and the majority of these were inappropriate, with higher rates in nonacademic clinics compared to academic clinics.
	Chandra Deb 2022^27^	USA	The necessity of antibiotic use in uncomplicated acute rhinosinusitis	Antibiotics were prescribed unnecessarily in 42.2% of encounters for indicated conditions.
	Gerber 2013^42^	USA	Effect of an AMS intervention on antibiotic prescribing for pediatric outpatients with acute respiratory tract infections.	Off-guideline antibiotic use for children with pneumonia decreased by 75% within 1-year post-intervention.
	Foreman 2022^28^	USA	Improvement of guideline-concordant antibiotic prescribing for urinary tract infections and skin and soft tissue infections.	There was no significant difference in appropriate dosing between pre-antimicrobial stewardship program and post- antimicrobial stewardship program groups. Safety-related patient outcomes did not differ between the groups.
	Frost 2022^29^	USA	Reduction in antibiotic prescribing for longer than institution-recommended durations for Acute Otitis Media in children aged 2 to 18 years.	The bundled intervention demonstrated superior improvement in guideline-concordant prescribing compared to the EHR-only approach.
	Giancola 2020^30^	USA	Adherence to guideline-recommended durations of therapy for uncomplicated cystitis in the outpatient setting.	Adherence to guideline-recommended directly observed therapy for uncomplicated cystitis increased and average duration of therapy prescribed decreased significantly post intervention
	Grigoryan 2021^25^	USA	Adherence to evidence-based practice guidelines for the treatment of uncomplicated cystitis.	Guideline-adherent prescriptions increased during the intervention period at both intervention and control sites.
	Hawes 2018^31^	Australia	Documentation of reasons for prescription for antibiotic prescription.	Reasons for antibiotic prescriptions were poorly documented, only in 82.7% of cases.
	Ivanovska 2018^32^	The Netherlands	National antibiotic prescribing guideline concordance.	Concordance to national antibiotic prescribing guidelines for pediatric fever, ear, and respiratory infections varied across different age groups.
	McCormick 2020^34^	USA	Appropriate choice of antibiotic based on first-line recommendations in guidelines: appropriate dose and appropriate duration of therapy.	Improvements in composite outcomes were primarily attributed to appropriate duration of therapy for the pharmacist-led intervention
	Owusu 2022^36^	Ghana	Compliance with standard treatment guidelines among adults with uncomplicated UTIs.	A high proportion of patient with uncomplicated UTI received empirical antibiotics. Of these, 90% of the antibiotic choices were guideline concordant. Only two-thirds received antibiotics for the recommended duration.
	Ray 2021^37^	USA	Antibiotic guideline-concordance for acute respiratory tract infections.	Telemedicine integrated into pediatric primary care practices improved guideline-concordant antibiotic management for acute respiratory tract infections.
	Robinson 2020^38^	USA	Appropriateness of empiric FQ use compared to nitrofurantoin for uncomplicated cystitis	FQs were commonly misprescribed for uncomplicated cystitis. Nitrofurantoin was deemed appropriate in 86.8% of cases, significantly higher than the 10.5% rate for FQ. Reasons for inappropriate fluoroquinolone use included absence of cystitis symptoms, FQ allergy, and recent non-susceptible urine culture. Inappropriate nitrofurantoin use was due to lack of cystitis symptoms, nitrofurantoin allergy, and prior non-susceptible urine culture. Appropriate FQ use was justified by nitrofurantoin intolerance or allergy, recent non-susceptible urine culture, suppressive therapy, or failed nitrofurantoin treatment.
	Vanstone 2022^40^	Canada	Documentation of indication when an antimicrobial is prescribed.	Antimicrobial indication documentation was improved with the audit and feedback intervention
Assessing patient and provider characteristics in prescribing	Brown 2019^26^	USA	Inappropriate prescribing between clinicians	Resident physicians had a lower rate of inappropriate prescriptions than attending physicians and advanced practice providers. Attending physicians issued 65.3% of all FQ prescriptions, with cystitis/pyelonephritis being the most common indication.
	Chandra Deb 2022^27^	USA	Comparison of individual provider rates of unnecessary prescribing based on their characteristics	Location, specialty, years of practice may influence prescribing; practitioners in rural settings and with more years in practice exhibited higher odds of unnecessary prescribing and providers in high-volume specialties like urgent care showed higher odds of unnecessary prescribing compared to those in family medicine.
	Grigoryan 2017^24^	USA	Relationship between patient characteristics and antibiotic prescribing for acute bronchitis.	Antibiotic prescribing was associated with patient’s age. While middle-aged adults (40–64 years) made the most visits for acute bronchitis, younger adults (18–39 years) had the highest antibiotic prescribing rates. Antibiotics were most frequently prescribed in the age group of 18 to 39 years, followed by 65 years and above, and 40 to 64 years. Mean duration of antibiotic prescription was 5.7 days for ages 18–39 years, 5.9 days for ages 40–64 years, and 6.0 days for ages ≥65 years. Macrolides were more commonly prescribed to younger adults, while fluoroquinolones were more common in older patients (≥65 years). Duration of antibiotic prescription was significantly longer in older adults. Sex and race were not associated with antibiotic prescribing.
	Hawes 2018^31^	Australia	Variations in antibiotic prescribing rates based on patient characteristics	Antibiotic prescribing rates per consultation varied significantly by age group. Patients aged 1–19 years received antibiotics at a higher rate per consultation compared to infants younger than 1 year and adults (over 19 years).
	Ivanovska 2018^32^	The Netherlands	Antibiotic prescribing patterns for children stratified by age, both in terms of degree of prescribing per diagnosis and choice of antibiotics	Adolescents received antibiotics more frequently than younger children despite fewer consultations. This was evident for both scenarios where antibiotics are recommended (pneumonia, strep throat, tonsillitis) or not recommended conditions (bronchitis, fever). For non-antibiotic-requiring diagnoses, adolescents had higher antibiotic prescription rates compared to younger age groups. Similarly, for antibiotic-requiring conditions, adolescents received more prescriptions than younger children. In children aged 0-4 years, amoxicillin was prescribed more frequently than in older age groups. Conversely, first-choice narrow-spectrum penicillin were more commonly prescribed in adolescents compared to younger children.
	Kitano 2021^47^	Canada	Association between total and unnecessary antibiotic use by family physicians, as well as to evaluate inter-physician variability in unnecessary antibiotic prescribing.	In unadjusted models, patient demographics correlate with higher unnecessary antibiotic prescribing rates; higher rates were observed in patients aged between 2 to 18 years, higher income, lower comorbidity, lower health utilization, and rural residence correlated. Physician factors including higher daily patient volume and non-US/Canada medical training are linked to increased unnecessary prescribing.
	Martínez-González 2020^49^	Switzerland	Patterns of antibiotic prescribing and its associated factors.	Women received more antibiotics than men across all clinical indications.
	Owusu 2022^36^	Ghana	Patient and prescriber characteristics associated with not being prescribed empirical antibiotics as recommended in the standard treatment guidelines.	Male patients were five times more likely to be prescribed a non-guideline-concordant antibiotic, with fewer than 10% receiving an antibiotic for the recommended duration.
	Robinson 2020^38^	USA	Predictors for FQ prescribing versus nitrofurantoin use in the treatment of uncomplicated cystitis.	Clinic of which the patient was treated at had the strongest influence on prescriptions. Increasing patient age, recent nitrofurantoin use within 90 days and a history of previous urine culture showing non-susceptibility to nitrofurantoin were also independent predictors of FQ use.
	Rockenschaub 2020a^21^	UK	The types of chronic obstructive pulmonary disease (COPD) patients get the most antibiotics.	The rate of first- and second-line antimicrobial therapies prescribed increased with the number of acute exacerbations during follow up. Patients who required hospitalization had lower rates of prescribing than those with three or more acute exacerbations of COPD (AECOPD) solely managed in primary care; however, these patients also had the highest usage of azithromycin. All three measures of COPD severity (FEV1, MRC scale and number of AECOPD at baseline) were associated with increased rates of antibiotic prescribing. Although they had the lowest rates of antibiotic prescribing (one to five prescriptions per year), patients with one or no record of AECOPD during follow-up received 56.1% of antibiotic prescribing after adjusting for age, sex, and social deprivation. Most of this prescribing was in patients with mild to moderate COPD, who still received 1.6 to 5.8 times as many antibiotics as the matched group without COPD.
	Rockenschaub 2020b^22^	UK	Association between the onset of comorbidity and antibiotic prescribing	Antibiotic prescribing rates rise significantly 4-9 months before COPD, heart failure, and asthma diagnoses, however this declines to baseline levels within 2 months post-diagnosis. A similar but less marked trend was observed for diabetes. This may suggest that respiratory symptoms may sometimes be misdiagnosed as respiratory tract infections which can lead to increased antibiotic prescribing. Rates of prescribing for patients with vascular conditions however, increased immediately before diagnosis but remained higher than baseline afterwards.
	Singer 2018^39^	Canada	Association between inappropriate antimicrobial prescribing and patient, prescriber, and practice-related factors.	Female patients, younger age, and fewer office visits were linked to potentially inappropriate antimicrobial prescriptions. Older patients, those with more comorbidities, more office visits, and visits to larger or rural practices were associated with potentially inappropriate antimicrobial prescribing.
	Sun 2019^23^	UK	Evaluate changes in antibiotic prescribing for different age groups, for male and female subjects, and by prescribing indication.	Variations by age and gender were observed, volume of antibiotic prescriptions were higher in very young and very old people and higher in women than men.
	Wang 2023^51^	China	Antibiotic prescription patterns among patients with ARIs in rural primary healthcare facilities	Female patients with ARIs were less likely to be prescribed antibiotics compared to male patients. Older patients were more likely to be prescribed parenteral antibiotics. Patients with chronic diseases were prescribed parenteral and multiple antibiotics at a higher rate.
	Wong 2023^52^	Canada	RTI antibiotic prescribing rate variations by rural/urban, deprivation and mode of visit	Rural settings had higher baseline prescribing rates.
Evaluating novel tools or measures	Lautenbach 2022^33^	USA	Effectiveness of an electronic data extraction algorithm in identifying inappropriate of antibiotic prescribing, antibiotic choice and duration.	Electronic algorithms for identifying inappropriate prescribing, antibiotic choice, and duration showed excellent test characteristics with high sensitivity and specificity compared to a manual chart review.
	Vernacchio 2022^53^	USA	Ability to define a novel measure to assess clinicians’ overall antibiotic prescribing	The Antibiotic Likelihood Index (ALI) which measures the proportion of encounters with an antibiotic prescription within six reason for visit categories accounting for over 80% of all antibiotic prescriptions in primary care pediatric practice can serve as a quality metric for antimicrobial stewardship in primary care.
Measuring specific conditions and outcomes	Gulliford 2020^46^	UK	Frequency of serious bacterial infections at family practices with lower antibiotic prescribing rates and whether patterns of medical coding were associated with the apparent occurrence of serious bacterial infection.	No evidence indicating that reduced antibiotic prescribing in family practices leads to higher rates of serious bacterial infections. A substantial portion of uncoded prescriptions includes repeat prescriptions, often for prolonged or serious infections.
	Loadsman 2019^48^	The Netherlands	Impetigo incidence, treatments and recurrence	Patients with impetigo often only have one or two visits to their GP from diagnosis to last contact and were treated satisfactorily with topical fusidic acid. Oral treatment was used in increasing frequency when second, or third treatments were given, with subsequent prescriptions usually written within 7 days. Patients experiencing multiple impetigo infections were more likely to be young children and/or suffering from eczema. A total of 124 episodes (7%) were recurrent within the same year.
	Moskow 2016^35^	USA	Documentation of beta-lactam allergies	Women had a higher documented beta-lactam allergy rate compared to men. White patients showed higher rates of documented beta-lactam allergy than black patients and those of multiple races. Hispanic patients were less likely to have a documented beta-lactam allergy than non-Hispanic patients. Skin rashes/hives accounted for 49.1% of all reported beta-lactam reactions. 36.2% of reactions lacked specific information
	Soudais 2021^50^	France	Prevalence of male UTIs in the general practice setting, diagnostic approaches and the prescribed treatments	Male UTIs are rare in the general practice setting. Fever was the sole clinical indicator significantly associated with prostatitis diagnosis. Over half of diagnoses lacked specific localization due to ambiguous clinical criteria distinguishing cystitis, pyelonephritis, or prostatitis. Less than 20% of UTI diagnoses were confirmed bacteriologically, with substantial missing data and methodological biases. Rectal examinations were infrequent in diagnostics, and urine dipstick tests were rarely used
	Wong 2023^52^	Canada	RTI antibiotic prescribing rates by rural/urban, deprivation, and mode of visit (in-person versus virtual)	Consultations for RTI dropped overall by 47.3% post-pandemic. In-person visits decreased by 37% and virtual visits increased correspondingly.

Among all the use categories, the most common were for analyzing prescribing trends and examining patient and provider characteristics related to antimicrobial prescribing. Large databases such as CPRD,^54^ NIVEL Primary Care Database,^55^ Secure Anonymised Information Linkage,^56^ Julius General Practitioners Network,^57^ and POLAR^58^ were used for this purpose. Individual practice and smaller-scale EMR data were used mainly for studies measuring the effectiveness of interventions, assessing antimicrobial prescribing quality, or evaluating specific conditions or outcomes.

### Strengths and facilitators

Analysis revealed several descriptions of strengths of EMR data for AMS across each of the assessed publications. These were grouped into three overarching categories, which were (i) the availability of comprehensive data where patient, encounter, and practitioner-level data (e.g. comorbidities, signs and symptoms, encounter reason, age, sex, race, allergies, diagnosis, sociodemographic details) and prescription details were captured sufficiently to enable assessments that facilitated assessment of prescribing quality and trends, measurements of intervention effectiveness and outcomes, and evaluation of patient or provider characteristics and novel tools;^19,20,22,24,25,33,35,36,38,42,50,53^ (ii) coded and standardized data which allowed effective identification of patients or conditions of interest,^24,25,27,30,33–37,45,46^ and (iii) large centralized databases with nationwide and longitudinal data which allows findings to be more broadly representative and likely more generalizable.^19,21–23,32,45,48,49,51^

Facilitators for effective EMR data use for AMS were: (i) availability of electronic prescriptions linked with EMR data enabling comparisons of dispensing rates and outcomes, and economic evaluations to be performed,^39,45^ (ii) automatic coding tools to improve the quality of data extracted, (iii) mandatory documentation of fields driven by financial incentives which contributes to improved data completeness,^53^ (iv) good EMR workflows for data entry which ensured cleaner and more complete data,^36^ (v) interoperability and data linkage between EHR systems and other databases,^21,29,42,43,46,47^ and (vi) established processes data access and collection.^23–25,29,31–33,45,52^

### Barriers and limitations

Descriptions of barriers were only included in a few studies. Barriers described were: (i) inconsistent EMR design across different systems leading to interoperability challenges, where standardization of data was required before use. Authors of one study concluded that “prescribing patterns can be influenced by system design”^28^ and in another “each EMR system has different architecture, even within one EMR system there may be province-specific differences in the EMR structure where information is stored”^52^ and (ii) cited “technical challenges in data extraction processes” leading to the exclusion of data from some regions.^52^

Descriptions of potential data quality issues were identified in 23 out of 34 studies. Data quality descriptions could not be found in four studies; the absence of this does not suggest that there were no data quality problems. Thirteen studies described elements of data completeness as limitations, such as (i) encounters without corresponding diagnostic codes.^47^ (ii) unlinked microbiology results^36^ and severity measures,^21^ or key clinical observations^38^ leading to less-comprehensive or unusable data for stewardship activities, (iii) absence of detailed records of the number of consultations causing problems in ascertaining whether an intervention was executed as intended,^41^ and (iv) missing data fields in historical records leading to potential inconsistencies in longitudinal data.^45^ Several studies highlighted the importance of mandatory documentation. One described poor allergy detail documentation where more than 36% (n = 13,679) of patients with a documented beta-lactam allergy failed to have any further description of their allergy, “making it difficult to know if the documented allergy is a true allergy and life-threatening (eg anaphylaxis); a known or anticipated, but undesirable, side effect (eg nausea); or a symptom of illness”.^35^ Indication was also described to be poorly documented in several studies;^22,23,31^ and were often captured in free-text in different places depending on the structure of the clinical software package used,^31^ leading to potential “underestimation of associations” where “the direction of bias cannot always be anticipated”.^46^ One study noted this to be particularly problematic for “antibiotic prescriptions based on telephone calls”.^38^.

Issues surrounding the plausibility of data (the believability or truthfulness of data values^59^) were described in fifteen studies. Several studies described unreliable, incomplete, or inaccurate documentation^22,28,38,46,48,51^ as potential issues, some reflected this through descriptions of manual review requirements to ensure accuracy.^28^ Other issues were (i) EMR system design limitations potentially discouraging accurate reporting of antimicrobial prescriptions or diagnoses leading to incomplete or biased data,^30,39,42^ (ii) inability to capture data regarding symptoms and comorbidities causing indication coding errors,^30^ (iii) broken links between consultation and antibiotic data necessitating certain assumptions to be applied before use e.g., if an antibiotic prescription did not link directly to a consultation, the previous or subsequent consultation in that year was used to determine the patient’s age at time of consultation,^31^ (iv) differences in coding between practices contributing to data inconsistencies,^44^ and (v) duplication in data entry through unstructured entries.^35^ More general descriptions of potential limitations relevant to data quality were also described in some studies such as: “limitations of the EHR”^28^, “possible missing data from external sources”,^20^ issues “inherent to the quality of the databases through standardization and data structuring”,^50^ and “limitations in the completeness of the GP records”^32^.

A summary of strengths, facilitators, barriers, and limitations for each study is presented in Table 2.

**Table 2.** Summary of identified strengths, facilitators, barriers, and limitations by study

Reference	Strengths and facilitators	Barriers and limitations
Adekanmbi 2020^45^	Availability of coded and/or standardized data available in a large, representative database which includes electronic prescriptions linked to EMR data and established processes for data access and collection.	Data completeness, no data recorded prior to 2013 making it impossible in some cases to assess for the presence of chronic conditions and smoking status.
Blair 2023^41^	Established processes for data access and collection.	Data completeness, unable to determine the actual number of consultations. “The absence of such information constrains our ability to assess whether the intervention was executed as intended and if its non-utilization impacted our findings.”
Brown 2019^26^	Electronic prescriptions linked to EMR data.	No details provided.
Chandra Deb 2022^27^	Availability of coded and/or standardized data.	No details provided.
Foreman 2022^28^	No details provided in study.	The authors concluded that prescribing patterns can be influenced by EHR design. Data quality limitations in completeness and plausibility were identified, data were not available for assessing compliance with order sentences due to “limitations of the EHR” and data accuracy was described to be dependent on external expert knowledge.
Frost 2022^29^	Established processes for data access and collection.	No details provided.
Gerber 2013^42^	Comprehensive patient- and visit-level data available including all prescriptions generated during a patient encounter and data linkage through a shared EHR where antibiotic orders are associated with encounter diagnosis codes.	Plausibility of data was a potential limitation, authors described that data may not reflect the actual situation.
Giancola 2020^30^	Availability of a centralized database with coded and/or standardized data.	Plausibility of data, where incorrect data may have been entered as there were possible errors in coding for complicated cystitis or pyelonephritis identified.
Grigoryan 2017^24^	Availability of comprehensive, coded and/or standardized data, and established processes for data access and collection.	Possible data plausibility issues as authors stated that they were unable to review patient symptoms documented in free text that may have influenced antibiotic prescribing decisions in some cases.
Grigoryan 2021^25^	Availability of comprehensive coded and/or standardized data which includes additional patient-level variables such as comorbidities, antibiotic allergies, type of antibiotic prescribed and duration of treatment, and established processes for data access and collection.	No details provided.
Gulliford 2019^20^	Availability of comprehensive, coded and/or standardized data.	Possible plausibility issues, authors state that it was possible for unreliable documentation for delayed or deferred prescriptions.
Gulliford 2020^19^	Availability of a comprehensive large, linked, and representative database with coded and/or standardized data.	Possible plausibility issues, no indication documentation leading to likely misclassification. Authors also state that nearly half of the antibiotic prescriptions were not associated with specific coded indications.
Hawes 2018^31^	Established processes for data access and collection.	Possible completeness issues, the data extracted were entirely dependent on the clinical software package and what general practitioners chose to document and where. The ‘reason’ field was free text and lacked standardization, authors also state that some links between the consultation data and the antibiotic data may have been inadvertently broken.
Ivanovska 2018^32^	Availability of a large, representative database, and established processes for data access and collection.	Authors state that there were limitations in the quality and completeness of the GP records.
Kitano 2021^47^	Linked database available where patient data were linked to multiple national databases such as the registered persons database, health insurance plan databases, and ambulatory care reporting systems, and established processes for data access and collection.	Authors state that the proportion of unnecessary antibiotics may be underestimated.
Lautenbach 2022^33^	Availability of comprehensive, coded and/or standardized data, and established processes for data access and collection.	Possible plausibility issues where the accuracy of the algorithm is dependent on clear documentation within the EMR.
Llor 2022^43^	Data linkage where records also included information on whether a prescription was filled.	No details provided.
Loadsman 2019^48^	Availability of a large, representative database.	Potential plausibility issues as there were limited access to the free text notes and access to information about the severity, extension of the symptoms and lesions, and the specific reason for a subsequent consultation.
Martínez-González 2020^49^	Availability of a large, representative database.	No details provided.
May 2022^44^	No details provided.	Potential plausibility issues as diagnostic coding practices may vary between clinics, and both chief complaints reported and those targeted for point-of-care testing may not have accurately captured all patients of interest.
McCormick 2020^34^	Availability of coded and/or standardized data.	No details provided.
Moskow 2016^35^	Availability of comprehensive, coded and/or standardized data, including a specific drug allergy module, and established processes for data access and collection.	Data completeness was described as an issue, 35% of drug allergy code fields were empty and “Among patients with a documented beta-lactam allergy, >36% of patients had no defined allergy reaction”.
Owusu 2022^36^	Availability of comprehensive, coded and/or standardized data. Cleaner and more complete data was possible due to thoughtful workflows, where indication documentation is performed at the time of entering clinical details.	Data completeness may be an issue, microbiology results were not available and not able to be accounted for in determining compliance with guidelines.
Ray 2021^37^	Availability of coded and/or standardized data.	No details provided.
Robinson 2020^38^	Availability of a centralized database with comprehensive data and good documentation of comorbidities.	Incomplete data, authors describe the following, “sometimes, records were incomplete or had missing data, particularly in the case of antibiotic prescriptions based on telephone calls. For example, some patients did not have a temperature documented, which was used to determine the presence of fever suggesting pyelonephritis and leading to study exclusion.”
Rockenschaub 2020a^21^	Existing data linkage to hospital admissions and census data, and established processes for data access and collection.	Data were incomplete for exposures such as the indication for the prescription and severity measures.
Rockenschaub 2020b^22^	Availability of a centralized data base with comprehensive data, and established processes for data access and collection.	Issues with completeness of data, information that may be relevant to the prescribing decision such as the reason for the prescription was not well recorded.
Singer 2018^39^	Data from electronic prescriptions available.	Potential plausibility of data issues as only one code can be entered per visit allowing the possibility for some granularity in a clinical presentation to be overlooked.
Soudais 2021^50^	Availability of comprehensive data and automatic coding tools which improved the quality of the data extracted.	Lack of data structuring may have led to information bias.
Sun 2019^23^	Availability of a large, representative database, and established processes for data access and collection.	Possible issues with data completeness, authors describe, “overall, more than half of the antibiotic prescriptions were documented without specific clinical conditions recorded.”
Vanstone 2022^40^	No details provided.	No details provided.
Vernacchio 2022^53^	Availability of comprehensive data and mandatory data fields driven financial incentives. “Reason for visit is a required element for insurance billing it is standard in all EHR systems and is included in the vast majority of encounters.”	No details provided.
Wang 2023^51^	Availability of a large, representative database.	Incomplete documentation: “antibiotic prescription rate of outpatient prescriptions may have been slightly biased after excluding prescriptions with missing diagnosis and/or drug regimen.”
Wong 2023^52^	Availability of a large, representative database, and established processes for data access and collection.	Inconsistent data architecture between EMR systems and technical challenges in data extraction.

## Discussion

AMS efforts such as ongoing surveillance, audit and feedback, and decision support are urgently needed in the primary care setting to address AMR and improve patient care. However, these programs are often not well-established compared to their secondary and tertiary counterparts. EMR data enables analysis of clinical data to be performed at scale to support AMS, relieving some of the additional human resource burden traditionally required to perform these activities. This review has provided evidence of how primary care EMR data have been used to aid with AMS and extracted strengths and facilitators of use, and barriers and limitations across different countries and settings.

EMR system design inconsistencies were the most commonly cited barrier among the reviewed studies suggesting an absence of a standardized approach in design across different vendors. “Technical challenges in data extraction processes” was also cited, emphasizing the need for improved technical infrastructure and data management practices. Issues of data completeness and plausibility were also commonly reported as limitations where key issues included potential negative impacts on patient care caused by poor documentation of allergies, and over-reliance on free-text data for data entry in EMR systems leading to implausible and/or unusable data.

Strengths and facilitators of EMR data included the availability of large centralized databases, comprehensive, linked, coded, and standardized data, facilitated by the implementation of mandatory documentation and standards, and automated coding tools for data extraction. Arguably, the most important facilitator identified was established processes for data access and collection through supportive regulation for data access and embedding data collection into standard practice. These approaches ensure timely access to the data necessary for AMS activities to be conducted efficiently and effectively.

A notable limitation of this review was that the methodology stipulated that articles published in languages other than English were to be excluded. The rationale for this was that authors felt that the accuracy and consistency of translation from software could not be guaranteed, especially for nuanced scientific and clinical content. That said, the initial database searches were not filtered by language, and yet, did not yield relevant non-English articles. Therefore, no publications were excluded based on language alone.

A glaring observation from this review was that the included publications were highly skewed towards high-income countries, with only one study from a low and middle-income country (LMIC). This is unsurprising for several possible reasons: (i) the ongoing phenomenon of under-representation of research literature in LMICs due to inequity in access to health systems research^60,61^ and (ii) the delayed uptake of EMR systems in LMICs due to infrastructure challenges, financial constraints, and the absence of resources required to maintain these systems.^62–67^ These inequities hamper the ability to perform AMS optimally and potentially further exacerbate the impacts of AMR where such countries are ironically expected to bear the heaviest consequences.^68,69^ Greater attention from the global community is required, and further efforts in capacity building, advocacy, and investment in infrastructure are urgently needed in these regions to ensure equity.

While this review highlights the importance of primary care EMR data as a useful resource for supporting AMS, equity in access to certain identified ‘strengths and facilitators’ such as large databases and automated coding tools are limited to higher-income settings. However, careful system design, effective data management practices, and supportive policies for reliable data access and collection processes, to overcome some of the identified barriers and limitations can still be implemented despite limited resources. Additionally, this review has proven that smaller-scale, high-quality EMR data, likely to be more assessable in most settings, are still extremely valuable for evaluating interventions and initiatives, and should continue to be the focus of investment to ensure effective AMS in the primary care setting.

## Supporting information

Cheah et al. supplementary material Cheah et al. supplementary material

## Supplementary material

For supplementary material accompanying this paper visit http://doi.org/10.1017/ash.2024.499. click here to view supplementary material
