# Supplementary material for: A systematic review on how primary care electronic medical record data have been used for antimicrobial stewardship
Source: Antimicrob Steward Healthc Epidemiol. 2025 Jan 24;5(1):e16. doi: 10.1017/ash.2024.499 (PMC11795443; doi:10.1017/ash.2024.499)
Supplement: Cheah et al. supplementary material [file S2732494X24004996sup001.docx]

**Supplementary**

**Table S1: Search Strategy**

1. **PubMed**

| Search | Concept | Search terms |
| --- | --- | --- |
| #1 | Primary Care | ("primary health care" OR "general practice" OR "community" OR "family practice" [MeSH Terms]) OR (“family medicine” [Text Word] OR “gp” [Text Word]) |
| #2 | Antimicrobial Stewardship | ("antimicrobial stewardship" OR "antibiotic stewardship" [MeSH Terms]) OR ("ams" [Text Word] OR "asp” [Text Word]) |
| #3 | Electronic Medical Record data | "electronic health record*" [MeSH Terms] OR "emr" [Text Word] OR "ehr" [Text Word] OR "electronic medical record*" [Text Word] |
| #4 | Use of primary care electronic medical record data for antimicrobial stewardship | #1 AND #2 AND #3 |
| Results: 17 | | |

1. **CINAHL**

| Concept | Search terms |
| --- | --- |
| Primary Care | ("Primary Health Care" OR "General Practice" OR "Community Health Services" OR "Family Practice") OR ("Family Medicine" OR "gp") |
| Antimicrobial Stewardship | ("Antimicrobial Stewardship" OR "Antibiotic Stewardship") OR ("ams" OR "asp") |
| Electronic Medical Record data | MH "Electronic Health Records" OR ("electronic health record*" OR "emr" OR "ehr" OR "electronic medical record*") |
| Use of primary care electronic medical record data for antimicrobial stewardship | (("Primary Health Care" OR "General Practice" OR "Community Health Services" OR "Family Practice") OR ("Family Medicine" OR "gp"))  AND (("Antimicrobial Stewardship" OR "Antibiotic Stewardship") OR ("ams" OR "asp")) AND (MH "Electronic Health Records" OR ("electronic health record*" OR "emr" OR "ehr" OR "electronic medical record*")) |
| Results: 26 | |

1. **Embase**

| Search | Concept | Search terms |
| --- | --- | --- |
| #1 | Primary Care | ('primary health care' OR 'general practice' OR 'community health care' OR 'family practice') OR (family medicine.mp. OR gp.mp.) |
| #2 | Antimicrobial Stewardship | ('antimicrobial stewardship' OR 'antibiotic stewardship' OR 'antimicrobial stewardship' OR 'antibiotic stewardship' OR 'ams' OR 'asp') |
| #3 | Electronic Medical Record data | ('electronic health record' OR 'electronic medical record' OR 'emr'.mp. OR 'ehr'.mp. OR 'electronic health record*'.mp. OR 'electronic medical record*'.mp.) |
| #4 | Use of primary care electronic medical record data for antimicrobial stewardship | #1 AND #2 AND #3 |
| Results: 56 | | |

1. **Scopus**

| Concept | Search terms |
| --- | --- |
| Primary Care | TITLE-ABS-KEY("primary health care" OR "general practice" OR "community health" OR "family practice" OR "family medicine" OR "gp") |
| Antimicrobial Stewardship | TITLE-ABS-KEY("antimicrobial stewardship" OR "antibiotic stewardship" OR "ams" OR "asp") |
| Electronic Medical Record data | TITLE-ABS-KEY("electronic health record*" OR "emr" OR "ehr" OR "electronic medical record*") |
| Use of primary care electronic medical record data for antimicrobial stewardship | ( TITLE-ABS-KEY ( "primary health care" OR "general practice" OR "community health" OR "family practice" OR "family medicine" OR "gp" ) ) AND ( TITLE-ABS-KEY ( "antimicrobial stewardship" OR "antibiotic stewardship" OR "ams" OR "asp" ) ) AND ( TITLE-ABS-KEY ( "electronic health record*" OR "emr" OR "ehr" OR "electronic medical record*" ) ) |
| Results: 104 | |

1. **Web of Science**

| Concept | Search terms |
| --- | --- |
| Primary Care | TS=("primary health care" OR "general practice" OR "community health" OR "family practice" OR "family medicine" OR "gp") |
| Antimicrobial Stewardship | TS=("antimicrobial stewardship" OR "antibiotic stewardship" OR "ams" OR "asp") |
| Electronic Medical Record data | TS=("electronic health record*" OR "emr" OR "ehr" OR "electronic medical record*") |
| Use of primary care electronic medical record data for antimicrobial stewardship | (TS=("primary health care" OR "general practice" OR "community health" OR "family practice" OR "family medicine" OR "gp")) AND (TS=("antimicrobial stewardship" OR "antibiotic stewardship" OR "ams" OR "asp")) AND (TS=("electronic health record*" OR "emr" OR "ehr" OR "electronic medical record*")) |
| Results: 62 | |

**Table S2. Terms for inclusion and exclusion**

| Terms highlighted for potential inclusion | Terms highlighted for potential exclusion |
| --- | --- |
| "human", "EHR", "EMR", "data quality", "antimicrobial stewardship", "AMS", "ASP", "appropriateness", "quality improvement", "stewardship", "health", "record", "primary", "medical", "guideline", "concordance", "compliance", "digital", "electronic", "records", "ambulatory", "outpatient", "community", "family" | "animal", "veterinary", "vet", "hospital", "systematic review", "meta-analysis", "meta-analyses", "hospitals", "emergency", "inpatient", "ED", "ICU", "ward", "wards", "inpatients", "scoping", "opioid" |
